# Multi-Source Causal Invariance for Cuffless Blood Pressure Estimation Based on Photoplethysmography Signal Features

**DOI:** 10.3390/s25113254

**Published:** 2025-05-22

**Authors:** Yiliu Xu, Zhaoming He, Hao Wang

**Affiliations:** 1 Research Center of Fluid Machinery Engineering & Technology, Jiangsu University, Zhenjiang 212013, China; 2 Department of Mechanical Engineering, Texas Tech University, Lubbock, TX 79411, USA; 3 School of Electrical and Information Engineering, Jiangsu University, Zhenjiang 212013, China

**Keywords:** cuffless blood pressure estimation, multi-source, feature selection, photoplethysmography, biomedical signals

## Abstract

Cuffless continuous blood pressure (BP) monitoring is essential for personal health management. However, its accuracy is challenged by the diversity and heterogeneity of physiological data sources. We propose a multi-source feature selection framework based on Markov blanket theory and the concept of causal invariance. We extracted 218 BP-related photoplethysmography (PPG) features from three heterogeneous datasets (differing in subject population, acquisition devices, and methods) and constructed a causal feature set using the Multi-Dataset Stable Feature Selection via Ensemble Markov Blanket (MDSFS-EMB) algorithm. BP estimation was then performed using four machine learning models. The MDSFS-EMB algorithm integrated PPFS and HITON-MB, enabling adaptability to different data scales and distribution scenarios. It employed Gaussian Copula Mutual Information, which was robust to outliers and capable of modeling nonlinear relationships. To validate the effectiveness of the selected feature set, we conducted experiments using an independent external validation dataset and explored the impact of data segmentation strategies on model prediction outcomes. The results demonstrated that the MDSFS-EMB algorithm has advantages in feature selection efficiency, prediction accuracy, and generalization capability. This study innovatively explores the causal relationships between PPG features and BP across multiple data sources, providing a clinically applicable approach for cuffless BP estimation.

## 1. Introduction

Cardiovascular diseases (CVD) are among the leading causes of mortality worldwide [1]. In non-clinical settings, blood pressure (BP) is commonly used as a key biomarker to assess cardiovascular status [2]. Studies suggest that BP variability, which reflects dynamic fluctuations in BP rather than its average value, has a more significant impact on the onset and prognosis of CVD [3]. Therefore, achieving continuous BP monitoring to capture these dynamic changes is of great clinical importance for the diagnosis and effective management of CVD.

The gold standard for continuous BP measurement is invasive intra-arterial monitoring, but its risks including bodily harm and infection, making it generally reserved for critical patients [4]. With advancements in flexible sensing technology and artificial intelligence, user-friendly and cost-effective cuffless continuous BP estimation techniques have emerged and become a central focus in continuous BP estimation research, overcoming the limitations of direct continuous BP measurement [5,6,7,8]. In particular, photoplethysmography (PPG) signals have gained significant attention due to their low cost and seamless integration into wearable devices. Recent studies leveraging feature selection and model fusion techniques based on PPG signals have also demonstrated the potential for PPG as an independent factor in BP estimation [9,10,11]. However, data-driven models often suffer from the “black box” limitation, lacking interpretability and robustness. The application of the causal inference method to construct the corresponding regression model is expected to break through the over-dependence on data correlation of the existing techniques and improve the accuracy of cuffless BP detection.

In contrast to traditional methods that rely on statistical correlations, causal feature selection reconstructs a subset of features by identifying the Markov Blanket (MB) of the target variable—comprising nodes with direct causal associations. When the faithfulness condition is satisfied, the MB reveals the local causal structure around the target variable and has been shown to be an optimal solution to the feature selection problem [12,13]. Unlike conventional approaches, causal feature selection not only filters out spurious correlations but also uncovers the intrinsic relationships between physiological mechanisms and BP characteristics. There have been studies applying causal relationships to the field of cuffless BP estimation [14,15,16]. Liu et al. were the first to introduce causal reasoning into cuffless BP estimation by constructing a causal graph between electrocardiography, PPG features and BP, which was used as an input for machine learning models to predict BP [15]. Building on this foundation, subsequent research integrated causal reasoning with graph neural networks, leveraging both the spatial information embedded in the causal graph and the temporal dynamics of heartbeat signals to enhance cuffless BP estimation [16]. However, these studies are limited to single datasets. Compared to single datasets, causal features identified across multiple datasets carry richer and more reliable causal knowledge, potentially improving the stability and cross-domain generalizability of cuffless BP estimation models [17].

This study aims to develop a multi-source causal feature selection framework that identifies PPG features with stable causal associations to build a generalizable cuffless continuous BP estimation model. We propose the Multi-Dataset Stable Feature Selection via Ensemble MB (MDSFS-EMB) algorithm, which is based on MB theory and the concept of causal invariance. The focus and main contributions of this work are threefold:

1. We propose a novel causal invariance-based feature selection framework for multi-source PPG data, ensuring the identification of stable and domain-invariant features;
2. MDSFS-EMB significantly reduces feature redundancy while retaining key causal features and enhanced robustness to device variations and population heterogeneity through nonlinear relationship modeling;
3. The MDSFS-EMB algorithm outperforms in feature selection efficiency, prediction accuracy, and generalization capability. External validation also further confirms that the causal feature set selected by the algorithm remains stable and reliable, even when applied to data with unknown distributions.

## 2. Materials and Methods

**Figure 1:**
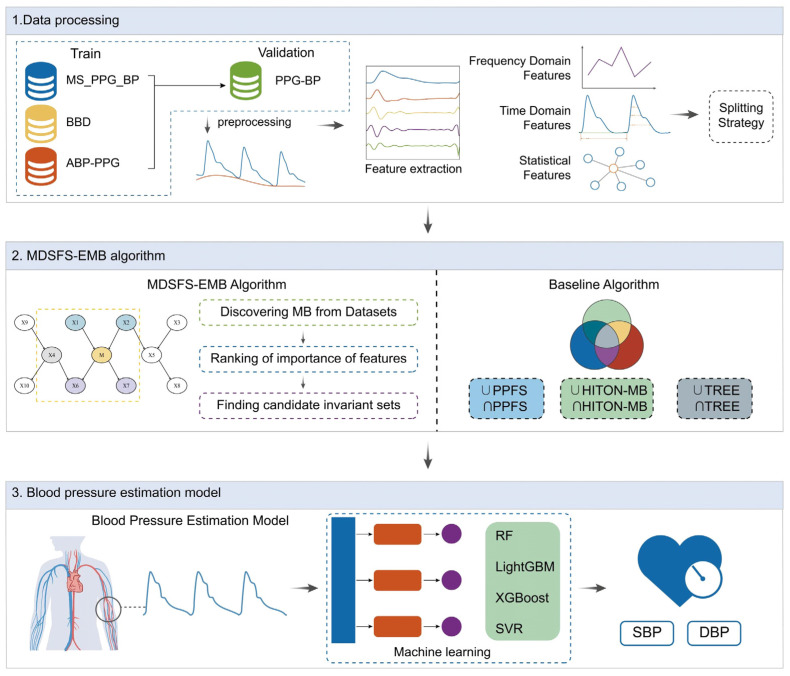
Experimental framework.

To construct a feature set applicable across multiple datasets and enhance the robustness of cuffless BP estimation models against external disturbances, we designed a multi-source dataset causal feature selection algorithm. As shown in Figure 1, the workflow of this experiment is as follows: Firstly, signal processing was performed, and features were extracted from the PPG signals. Next, the MDSFS-EMB algorithm was employed for feature selection. Finally, the identified causal feature set was used as input to build a cuffless BP prediction model. Additionally, this study examined the impact of segmentation strategies on model prediction outcomes.

### 2.1. Datasets

**Figure 2:**
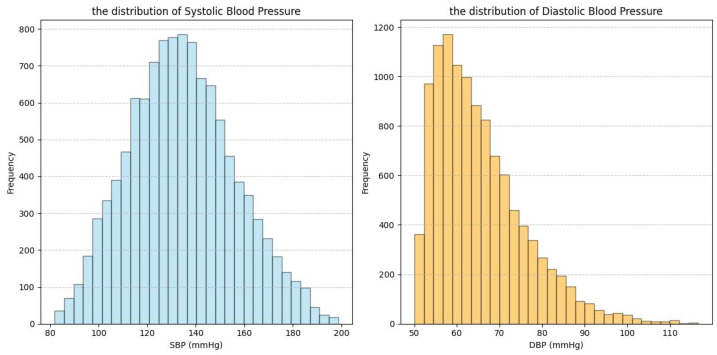
Data distribution graph for ABP_PPG dataset.

**Figure 3:**
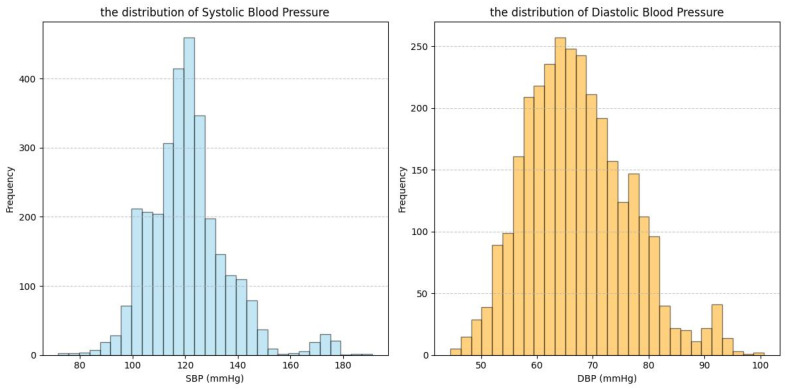
Data distribution graph for BBD dataset.

**Figure 4:**
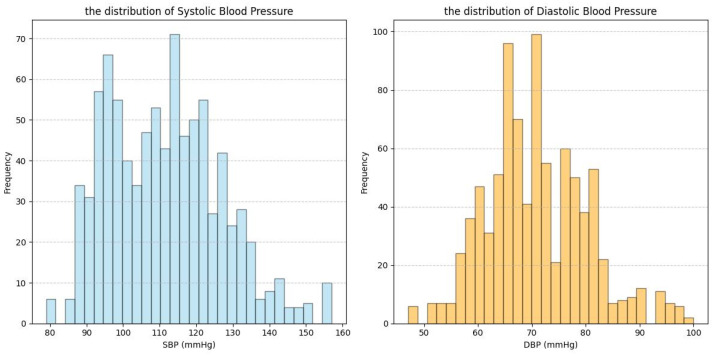
Data distribution graph for MS_PPG_BP dataset.

**Figure 5:**
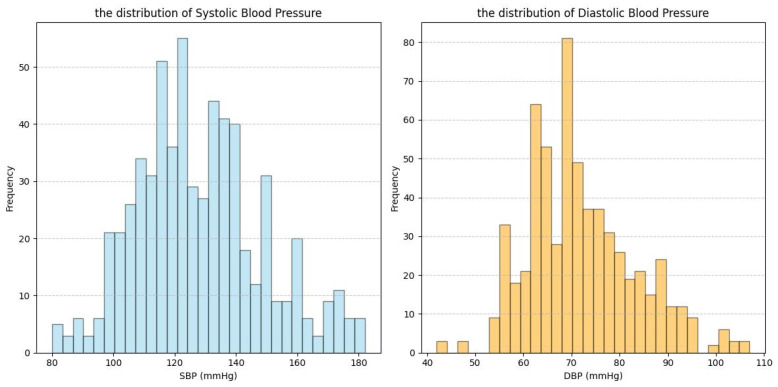
Data distribution graph for PPG_BP dataset.

This section describes the datasets used in this study: our Multi-state PPG BP Dataset (MS_PPG_BP) and three publicly available datasets—ABP_PPG [18], Ballistocardiography Dataset (BBD) [19], and PPG-BP [20]. ABP_PPG, BBD, and MS_PPG_BP were used for feature selection, while PPG-BP was used for validation of the causal feature set. Table 1 and Figure 2, Figure 3, Figure 4 and Figure 5 summarize the dataset information and BP distributions. The ABP_PPG dataset (from MIMIC-III [20]) comprised 15 s segments recorded every 5 min from 1195 patients (715 males) with PPG signals and arterial BP (ABP) waveforms. The BBD dataset recorded PPG and ABP signals from 40 subjects (17 males), characterized by lower variability and a narrow BP distribution. The PPG-BP dataset included recordings from 219 patients (105 males) with various cardiovascular conditions but fewer segments.

To capture BP changes induced by external interventions, we constructed the MS_PPG_BP dataset. The dataset contained multimodal data from 30 healthy subjects (18 males) in seven different physiological states. All subjects fulfilled the following conditions: no history of cardiovascular disease; no antihypertensive medication in the last two weeks; caffeine-based beverages were abstained from 24 h before the experiment; and they maintained a normal routine the day before the experiment. In the data acquisition process, for blood pressure measurement, an upper-arm electronic sphygmomanometer (OMRON U703, OMRON Industrial Automation (Dalian), Dalian, China) was used to measure the subject’s blood pressure on the right arm, two measurements were taken during each intervention, and the mean value was taken as the final data. The acquisition of PPG signals was accomplished with an infrared pulse sensor (HKG-07C, Hefei Huake Electronic Technology Research Institute, Hefei, China), which was capable of continuously collecting blood flow signals from the end of the subject’s right index finger at a sampling rate of 200 Hz, and the sensor was secured with medical tape to ensure stable and pressure-free contact.

The entire experimental procedure was divided into three phases. Firstly, there was the baseline period, in which the subjects sat still for 10 min and then measured the blood pressure in the left arm three times consecutively (with 2 min intervals), and the formal experiment was started only when the difference between the three measurements was <1 mmHg. This was followed by the intervention period, in which subjects were sequentially tested in seven states, including resting, grip strength, deep breathing, mental arithmetic, and dropping the hand in the sitting position, as well as resting and dropping the hand in the standing position, and resting and dropping the hand in the supine position, with each state lasting for 1 min, and with at least a 5-min interval between adjacent states, until the subject’s BP returned to the baseline level. Finally, there was a quality control session, where the researchers monitored the signal quality in real time, and when motion artefacts were found to be more pronounced in the signal, the subjects were asked to repeat the test for that state to ensure the reliability and validity of the data.

### 2.2. Data Processing and Feature Extraction

The signal preprocessing framework comprised three stages: (1) Resampling the PPG and ABP signals to 125 Hz and achieving signal alignment through the maximum mutual correlation algorithm; (2) Denoising using discrete wavelet decomposition, combined with the baseline drift correction technique: using cubic spline interpolation, the starting point of the pulse waveform was selected as the interpolation point to fit the baseline, and the baseline drift value was subsequently subtracted from the original PPG signal. The baseline adjustment was completed by correcting the PPG valley values; (3) The preliminary screening utilized signal quality index (SQI) and waveform feature thresholds. Signal quality was assessed using the skewness SQI (SQI-skew), which evaluated signal distribution symmetry, and the kurtosis SQI (SQI-kurtosis), which indicated data concentration around the mean. Subsequently, physiological plausibility checks were conducted, restricting blood pressure values to 40–200 mmHg and pulse pressure to a minimum of 10 mmHg. Physiological plausibility checks were then performed, limiting blood pressure values to the range of 40–200 mmHg and pulse pressure to no less than 10 mmHg, while quantitative analyses of peak-to-valley intervals and amplitude variations in the PPG patterns were performed to exclude signal passages that exceeded the statistical thresholds. Label construction varied for different datasets: for data containing PPG and arterial blood pressure waveforms, systolic and diastolic labels were extracted from each ABP segment by calculating the median systolic peak value and the median values of the start and end points of the cardiac cycle; whereas for datasets containing only the PPG signals and the blood pressure values, the preprocessed PPG signals were used directly and associated with the corresponding systolic and diastolic blood pressure values as labels.

Building on the PPG features extracted by Jishnu Dey et al. [21], this study derived a total of 218 features from the PPG signal and its first four derivatives—namely, velocity photoplethysmography (VPG), acceleration photoplethysmography (APG), jerk photoplethysmography (JPG), and snap photoplethysmography (SPG). These features encompassed frequency-domain, time-domain, and statistical characteristics, as detailed in Table 2.

**Figure 6:**
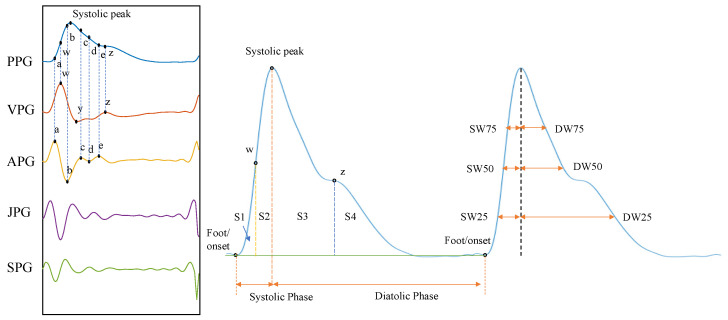
Selected time-domain features extracted from PPG signals and their derivatives.

Frequency-domain features were extracted by Fourier Transform (FFT), reflecting the frequency energy distribution of the signal, including the amplitude and frequency of the main peak of the signal in the frequency domain, as well as the average amplitude of the neighborhood. Time-domain features were used to assess cardiovascular function by analyzing points of interest and morphological parameters in the PPG and multistep derivative waveforms. The points of interest included the steepest rise (w), the maximum negative slope (y), and the diastolic rise (z) of VPG, and five feature points (a, b, c, d, e) of APG. The feature extraction covered four aspects: the PPG/VPG/APG amplitude at each point of interest, the inter-event interval (e.g., total cycle duration Tc), widths of the systolic and diastolic phases (SW and DW) at 25%, 50%, and 75% of the systolic peak amplitude, and the area under the segmental curve (S1–S4). Temporal features were normalized by dividing by Tc, and area features were normalized by the proportion of the total area. Figure 6 illustrates a selection of time-domain features extracted from the PPG signal and its derivatives. Statistical features included signal quality index (SQI-skew, SQI-kurtosis), slope deviation curves, exponential features, and histogram features. Exponential features included Aging Index, *I_bd_*, *I_bcda_*, and *I_sdoo_*, which supported the assessment of vascular aging and screening for atherosclerotic disease [22]. Slope deviation curves included the rising/descending slope deviation curves (USDC/DSDC): USDC was defined as the deviation of points along the rising edge from the mean rising slope, and DSDC corresponded to the deviation of points along the descending edge from the mean descending slope, and was used to quantify the relative speed of systolic and diastolic activity of the heart.

### 2.3. Feature Selection

#### 2.3.1. Causal Model

**Definition** **1.** *Faithfulness. A Bayesian network {V,G,P} is said to satisfy the faithfulness condition in a Bayesian network {V,G,P} if the conditional independence relation implied by the joint distribution P of all its variables is identical to the conditional independence relation expressed by the directed acyclic graph G. Moreover, if G is faithful to P, then P is faithful to G.*

**Definition** **2.** *MB. For a DAG G, the Markov condition holds in G if and only if each node of G is independent of any subset of nondescendants conditioned on its parent, e.g., in random variables U of full set, for a given variant* *X* *∈* *U and set of variables* *MB* *⊂* *U(X* *∉* *MB), if*

(1) $$X⊥\left\{U-MB-\left\{X\right\}\right\}|\;MB,$$

*then the set of variables MB that can satisfy the Markov condition is the MB of X.*

**Definition** **3.** *Causal Invariance. Causal invariance refers to the fact that certain causal relationships remain consistent under different environments or data distributions. Specifically, if the relationship between a set of features S and a target variable M is consistent across multiple datasets, then the relationship can be considered causally invariant.*

#### 2.3.2. MDSFS-EMB Algorithm

Traditional feature selection methods are typically designed for single datasets, and their direct application to multiple datasets may yield unreliable results. The independent selection of features followed by their intersection may lead to exclusion of critical biomarkers owing to data variability and inter-subject heterogeneity, whereas union-based aggregation strategies might incorporate redundant features that could elevate model overfitting potential. To address this issue, the study proposed the MDSFS-EMB algorithm. By incorporating the concept of causal invariance, the algorithm aimed to identify features that maintained a stable causal relationship with the target variable (BP) across different datasets, thereby enhancing the accuracy and stability of BP estimation. The pseudocode for the MDSFS-EMB algorithm is as follows: 

The MDSFS-EMB algorithm used two causal feature selection algorithms, PPFS [22] and HITON-MB [23], respectively, for each dataset to construct a feature subset.

- PPFS: Cross-validation based aggregation method to find MB for small samples and for datasets that do not follow the fidelity assumption;
- HITON-MB: MB-based local causal structure learning approach focusing on direct cause/effect identification and MB discovery. It is suitable for large-scale datasets, but may face challenges with small sample sizes.

This algorithm built upon the principles of MCFS [24] and MCRFS [17], which transformed the multi-source causal feature selection problem into a search for invariant sets by screening for a subset of features that keep the conditional probability distribution of the class attributes stable across all datasets: the stability of the feature set was ensured by using the average mutual information (AMI) as a criterion for selecting the invariant set, and t-tests were used to determine whether the mutual information of each dataset was significantly different from the AMI.

In order to be able to detect nonlinear causal relationships between BP and PPG features, this study utilized Gaussian Copula Mutual Information (GCMI) for the computation of mutual information [25]. GCMI preserved the rank relationships between variables by transforming the marginal distributions into standard normal distributions, thus providing a comprehensive measure of linear and nonlinear dependencies. The calculation formula is the following:

(2) $$I(X;Y)=\frac{1}{2}\log(\frac{\left|R_{X}|\cdot |R_{Y}\right|}{\left|R_{XY}\right|}),$$

$R_{X}$ is the covariance matrix of X, $R_{Y}$ is the covariance matrix of Y, $R_{XY}$ is the covariance matrix of the joint variable (X,Y), | ⋅ | denotes the determinant of the matrix, and log is the natural logarithm.

The algorithm compensated for the increased computational effort of GCMI by implementing multilevel optimization strategies: ranking candidate features according to mutual information and prioritizing high-importance features to reduce the combinatorial search space; limiting the maximum size of the subset to avoid combinatorial explosion; accelerating the process of hypothesis testing by using parallel computation; storing pre-stored values of mutual information to reduce the repetitive computation; and introducing dynamic pruning to terminate the redundant search in advance when enough valid combinations were found (See Algorithm 1).
**Algorithm 1.** The MDSFS-EMB Algorithm**Input:** D = {$D_{1},D_{2},\ldots ,D_{K}$}, M: BP, α: significance level, n: feature subset size**Output:** Best_feature**Step1:** Discovering MB from D*maxMB(M) = *∅;* minMB(M) = *∅;* supMB(M) = *∅3.**for** *i = 1 ***to** *K ***do**4.  //Find ${MB}_{H_{i}}$(M) and ${MB}_{P_{i}}$(M) in datasets $D_{i}$ with the HITON-MB and PPFS algorithms5.      ${MB}_{i}$*(M) =* ${MB}_{H_{i}}$*(M)* ∪ ${MB}_{P_{i}}$*(M)*6.      maxMB(M) = maxMB(M) $\cup \;{MB}_{i}$(M);7.      minMB(M) = minMB(M) $\cap \;{MB}_{i}$(M);8.supMB(M) = maxMB(M) − minMB(M);9.**Step2:** Ranking of importance of features10.  **for** *f* **in** *supMB* **do**11.        MI_avg(f) = 1/K × Σ compute_mutual_info($D_{i}$, M, f)12.supMB_sorted = sort(supMB, MI_avg, descending) // Sort by AMI13.**Step3:** Finding candidate invariant sets14.  **for** *subset_size **=** 1* **to** *n* **do**15.        **for** *S* **in** *candidates(subset_size, supMB_sorted)*16.            all_passed = True17.              **for** *i = 1* **to** *K* **do**18.                 MI_values = [compute_mutual_info(D_i, M, f) for f in S]19.                 p = ttest_1samp(MI_values, MI_avg(S))20.                 **if** *p <* α:21.                  all_passed = False22.                  **break**23.              **if** *all_passed:*24.                 Best_feature = S25.          **return** Best_feature // Return the first valid subset

### 2.4. BP Estimation

In machine learning model evaluation, dataset segmentation strategies are crucial for performance evaluation [26,27]. The segmentation strategies for BP datasets can usually be categorized into record-level splitting and sample-level splitting. To address the characteristics of the BP dataset with multiple records, strong individual dependency and skewed distribution of BP values, this study adopted record-level splitting to ensure that all samples from the same subject appear in only a single subset of the training, validation or test set. This strategy simulated the model’s ability to generalize to new subjects in real clinical scenarios by eliminating the risk of cross-individual information leakage. In contrast, sample-level splitting, although it can improve data utilization, may lead to cross-contamination of individual data and imbalance in the distribution of subset samples, which may introduce assessment bias. In this study, classical support vector regression (SVR), random forest (RF), Light Gradient Boosting Machine (LightGBM) and Extreme Gradient Boosting (XGBoost) were selected to construct a regression model for cuffless BP estimation. A five-fold cross-validation approach was used to separate training and validation sets for optimal model selection.

## 3. Results

### 3.1. Model Evaluation

The Association for the Advancement of Medical Instrumentation (AAMI) has developed specifications that require a mean error of prediction (ME) of ≤5 mmHg and a standard deviation of error (SDE) of ≤8 mmHg [28]. The IEEE 1708 standard further specifies that the mean absolute difference (MAD) needs to be controlled at ≤6 mmHg [29]. Mean Absolute Scaled Error (MASE) is a scale invariance metric that helps to comprehensively assess the accuracy and consistency of BP estimation models. The formulas for MAD, ME, SDE, and MASE are shown below:

(3) $$ME=\frac{1}{n}\sum_{i=1}^{n}({\hat{y}}_{i}-y_{i})$$

(4) $$MAD=\frac{1}{n}\sum_{i=1}^{n}\left(|{\hat{y}}_{i}-y_{i}\right|)$$

(5) $$STD=\sqrt{\frac{1}{n}\sum_{i=1}^{n}{\left({\hat{y}}_{i}-y_{i}\right)}^{2}}$$

(6) $$MASE=\frac{MAD}{MAD_{Naive}}$$

${\hat{y}}_{i}$ represents the model-predicted BP, $y_{i}$ denotes the actual BP value, *i* is the prediction index, *n* refers to the number of data points used for model prediction, and $MAD_{Naive}$ represents the median value of the BP of the training set.

### 3.2. Splitting Strategy

This study evaluated the effect of two different data splitting strategies on the performance of BP prediction models. The BDD dataset was used to predict BP by the LightGBM model. As shown in Table 3, for diastolic blood pressure (DBP) and systolic blood pressure (SBP) prediction, the prediction results of sample-level splitting were better than record-level splitting in all metrics. The predictions of sample-level splits for both pairs of DBP and SBP are compliant with the AAMI standard and the IEEE 1708 standard, while only the predictions of DBP for record-level splits are compliant with the standard.

**Figure 7:**
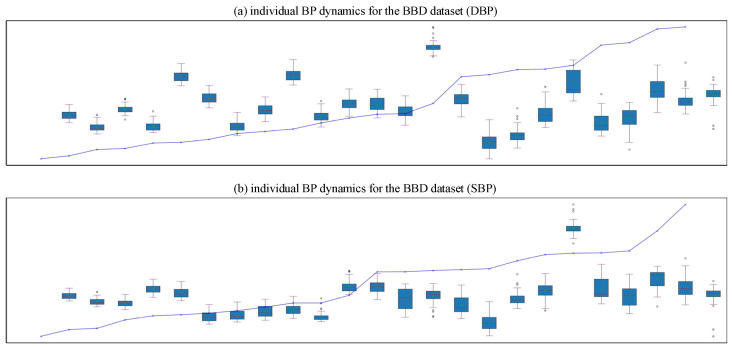
The results of record-level splitting applied to the BBD dataset: (**a**) Individual DBP dynamics—each record’s DBP distribution is represented using a box plot. The records are sorted in ascending order based on the difference between maximum and minimum DBP values; (**b**) Individual SBP dynamics.

**Figure 8:**
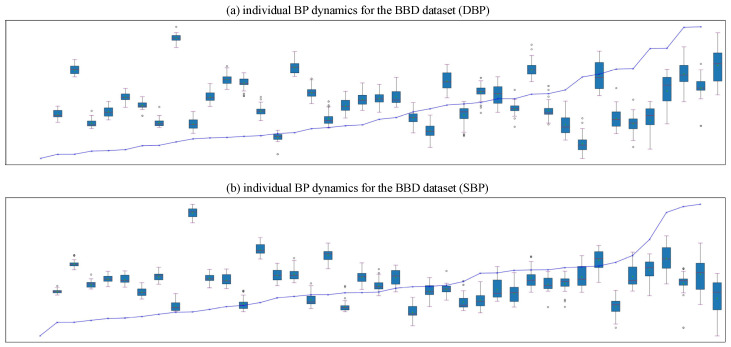
The results of sample-level splitting applied to the BBD dataset: (**a**) individual DBP dynamics; (**b**) individual SBP dynamics.

Individual BP dynamics was used to demonstrate the BP dynamics of each individual and to verify the model’s ability to adapt to individual differences. The distribution of outliers in Figure 7 was more concentrated, appearing earlier on the left side of the data with a smaller overall number, compared with Figure 8. The distribution of the data was relatively consistent, the trend of BP changes was relatively smooth, and there were no obvious signs of overfitting.

### 3.3. Feature Selection Results

**Figure 9:**
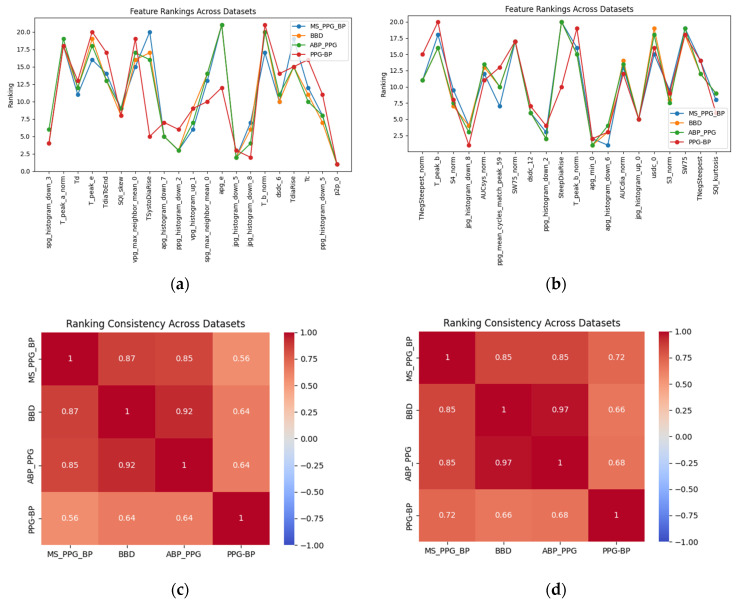
(**a**) Ranking of DBP features; (**b**) ranking of SBP features; (**c**) consistency matrix of DBP; (**d**) consistency matrix of SBP.

We ranked causal features separately for different datasets and assessed the consistency of feature rankings using Kendall’s tau correlation coefficient, as shown in Figure 9. The results showed that feature importance rankings are similar across datasets, indicating their robustness. Specifically, the selected feature set exhibited high consistency across the ABP_PPG, BBD, and MS_PPG_BP datasets, with good consistency observed in the external validation dataset as well. Furthermore, for DBP prediction, the chosen features primarily focused on diastolic hemodynamics and vascular elasticity, while for SBP prediction, the selected features emphasized systolic pressure propagation and cardiac contractility.

In order to further validate the effectiveness of the feature set, HITON-MB, PPFS and the Tree-based Ensemble method were used for feature selection in this study. According to Algorithm 2, baseline algorithm, the intersection or concatenation of features from each dataset were used as the final feature set, which was then compared with the feature set constructed by the MDSFS-EMB algorithm, as shown in Table 4.

The Tree-based Ensemble method calculated feature importance used the Gini coefficient by averaging the reduction in Gini impurity across all tree splits, normalizing these values, and ranking features based on their contribution to model prediction.
**Algorithm 2.** Baseline Algorithm**Input:** D = {$D_{1},D_{2},\ldots ,D_{K}$},M: SBP or DBP **Output:**
∪MB(M), ∩MB(M)
**for** *i = 1 ***to** *K ***do**    //Find ${MB}_{i}$*(M)* in datasets $D_{i}$ with the feature selection algorithm  **end**  ∪MB(M) = $\cup_{i=1}^{n}{MB}_{i}$*(M)*  ∩MB(M) = $\cap_{i=1}^{n}{MB}_{i}$*(M)*

Table 4 demonstrates that the MDSFS-EMB algorithm exhibited significant advantages in causal feature selection for multi-source PPG data. Compared to benchmark algorithms, the MDSFS-EMB algorithm achieved superior performance in both DBP and SBP prediction. Under the IEEE 1708 standard, its compliance rate reached 50%, significantly outperforming baseline methods (16.7–33.3%). Specifically, the MDSFS-EMB algorithm selected only 21 and 20 features for DBP and SBP tasks, respectively, which was considerably fewer than union-based approaches (∪PPFS for DBP used 105 features, and ∪TREE for DBP used 120 features). For DBP prediction, the MDSFS-EMB algorithm had lower average MAD values (5.24) than the other algorithms. For SBP prediction, the MDSFS-EMB algorithm also outperformed the other algorithms in terms of average MAD values (7.48). Moreover, on the external PPG-BP dataset, the MDSFS-EMB algorithm ranked second in DBP prediction error and first in SBP prediction error. Additionally, its scale-invariance metric MASE (SBP/DBP = 0.523/0.630) improved by 12.8–23.9% over the best baseline (∩TREE = 0.600/0.829) in external validation. The algorithm’s performance on the external validation dataset (PPG-BP) confirmed that its prediction error improvements were statistically significant.

### 3.4. Performance Comparison of Various Machine Learning Models

Feature sets from the MDSFS-EMB algorithm were fed into SVR, RF, LightGBM, and XGBoost models to compare performance across different datasets. Table 5 showed the performance of these machine learning estimation algorithms. To ensure fairness, all models were evaluated on standardized datasets—with uniform preprocessing, feature extraction, and evaluation criteria.

LightGBM exhibits the most balanced performance across all datasets, consistently obtaining low MAD and STD values for DBP and SBP predictions across multiple datasets, maintaining low errors and high stability. XGBoost excelled in specific tasks (e.g., MS_PPG_BP-DBP, PPG-BP-SBP), but its overall stability requires further assessment based on STD values. RF maintained stable performance in most datasets (e.g., PPG-BP-DBP, MS_PPG_BP-SBP), making it a suitable choice for bias-sensitive scenarios. While SVR performed relatively well in some SBP prediction tasks, it showed weaker and more volatile performance for DBP estimation. Overall, LightGBM stands out as a stable and accurate choice. Comparing the results with the AAMI and IEEE 1708 standards, most models met the criteria for DBP prediction across all datasets except ABP_PPG. However, for SBP estimation, only the MS_PPG_BP dataset using RF, LightGBM, and XGBoost produced results that complied with the standards. DBP predictions also achieved higher accuracy than SBP predictions.

## 4. Discussion

In this study, we designed a multi-source dataset causal feature selection algorithm to identify a consistent causal feature set across multiple datasets, addressing the challenge of cuffless BP estimation in diverse data environments. We extracted features from PPG signals, applied the MDSFS-EMB algorithm for feature selection, and built a causal regression model for cuffless BP estimation using machine learning methods. The constructed causal feature set demonstrated superior overall performance on the ABP_PPG, BBD, MS_PPG_BP, and PPG-BP datasets, outperforming the direct use of feature selection algorithms based on intersection and union strategies.

### 4.1. Advantages of the MDSFS-EMB Algorithm

Here, we pioneered the application of a multi-source causal feature selection algorithm to the field of cuffless BP estimation. By designing the MDSFS-EMB algorithm, we constructed a multi-source invariant feature set, successfully balancing detection rate and accuracy. Compared to baseline feature selection methods, the MDSFS-EMB algorithm demonstrated significant advantages in feature redundancy control and model generalization. While union-based methods maintained high recall rates, they introduced substantial redundancy (e.g., ∪TREE selects up to 120 features), which not only increased computational complexity but also raised the risk of overfitting due to irrelevant features. Conversely, intersection-based methods enforced overly strict feature selection criteria (e.g., ∩HITON-MB results in an empty feature set), ensuring high precision but at the cost of losing critical features, ultimately leading to reduced cross-dataset prediction stability. By leveraging causal invariance, the MDSFS-EMB algorithm effectively filtered out features that exhibited correlation in specific datasets but lacked genuine causal significance. The selected features remained consistently predictive across multiple datasets, making them more aligned with the true causal relationships. Experimental results indicated that MDSFS-EMB reduces computational overhead by 83% (compared to ∪TREE) while maintaining high prediction accuracy. The selected feature set exhibited high generalization in multi-source BP estimation. This combination of low complexity and high robustness makes MDSFS-EMB particularly well-suited for real-time BP monitoring in resource-constrained devices, such as wearable sensors.

As shown in Table 4, the proposed MDSFS-EMB algorithm has been thoroughly validated for its effectiveness in multi-source scenarios. Not only did it significantly outperform baseline algorithms, but it also demonstrated stable feature representation even across datasets with substantial differences. This validated the effectiveness of the causal feature set selected by the MDSFS-EMB algorithm, which remained reliable even for datasets with unknown distributions. We attribute its strong performance to three key factors:

1. By integrating the small-sample adaptability of PPFS with the large-scale structure discovery capability of HITON-MB, the algorithm overcomes sensitivity to dataset size while leveraging MB theory to comprehensively identify causal features of the target variable;
2. By transforming marginal distributions into Gaussian form, GCMI preserves rank relationships between variables, effectively capturing the nonlinear and non-monotonic associations between PPG signals and BP parameters. This approach not only enhances robustness to outliers but also eliminates distributional assumptions, making it highly adaptable to heterogeneous multi-source data;
3. GCMI- and AMI-based assessment mechanisms mitigate the impact of dataset heterogeneity on feature stability, ensuring consistent and reliable causal feature selection across different data sources.

In conclusion, the MDSFS-EMB algorithm demonstrates significant advantages in feature selection efficiency, prediction accuracy, and generalization ability. Not only does it exhibit low computational complexity and high robustness, but it also adapts well to datasets with different distributions. Future research could explore hybrid architectures integrating the MDSFS-EMB algorithm with deep learning, enabling the model to tackle more complex prediction and modeling challenges.

### 4.2. Data Heterogeneity Versus Model Generalization

The heterogeneity of multi-source data not only provided an opportunity to test the robustness of the algorithm but also presented challenges for improving model performance. The four datasets involved in this study exhibited significant differences in subject characteristics (e.g., patients vs. healthy young individuals), data quality (e.g., noise levels, collection protocols), and BP distribution (e.g., range, skewness) (Table 1, Figure 2, Figure 3, Figure 4 and Figure 5). While this diversity enhanced the validity of the validation framework, it also subjected the model’s prediction stability to multiple stress tests. Specifically, Table 5 showed the ABP_PPG dataset presented the greatest difficulty in prediction, which not only had the broadest BP range (SBP: 81.83–198.66 mmHg, DBP: 50.07–116.64 mmHg) but also exhibited significant individual variation in BP levels and patterns of change. BP values are influenced not only by dataset statistical features (e.g., BP range, distribution) but also by demographic factors (e.g., gender, age), health conditions (e.g., obesity, CVD), and other factors [30,31]. In particular, the ABP_PPG dataset comprised ICU patients, where medical interventions such as medications and surgeries are key external factors influencing BP variability. In contrast, the MS_PPG_BP dataset yielded the best prediction results with both DBP and SBP predictions meeting clinical standards in terms of MAD, STD, and ME values. This was attributed to the target population for the MS_PPG_BP dataset consisting primarily of healthy young individuals, with a narrower BP distribution range (SBP: 79–157 mmHg, DBP: 47–100 mmHg) and higher data quality, resulting in lower prediction difficulty.

The “double-edged sword” effect of the data splitting strategy further amplifies the complexity mentioned above [26,32]. On one hand, sample-level splitting, while improving data utilization through random assignment, may introduce data leakage due to overlap in individual records. On the other hand, although record-level splitting strictly isolates individual data and is more aligned with clinical scenarios, it can increase prediction errors due to insufficient coverage in the training set, which exacerbate the impact of individual differences. Therefore, we recommend that in clinical scenarios exhibiting significant intersubject variability and stringent safety requirements, record-level data partitioning should be prioritized to preserve evaluation authenticity and clinical reliability. For datasets demonstrating strong homogeneity where optimal utilization of limited samples is essential, sample-level splitting may be employed. However, it should be accompanied by k-fold cross-validation and rigorous leakage detection to mitigate potential risks.

The interaction between BP physiological characteristics and algorithm limitations also requires close attention. The wider dynamic range of SBP and its greater sensitivity to external factors such as peripheral vascular resistance contributed to the higher complexity of SBP prediction compared to DBP. In addition, the skewed distribution of BP values led to sparse samples for extreme values, making it difficult for the model to learn such patterns from limited data. Although the MDSFS-EMB algorithm alleviated this issue to some extent by causal feature selection, the multi-source shared feature selection strategy may overlook key covariates for specific populations (such as patients with CVD), like medication intervention records, which further limited the ability to capture extreme values. Future research should explore dynamic feature weighting mechanisms, which could enhance modeling of long-tail distributions while preserving causal invariance.

### 4.3. Limitation

This study also has several limitations:

- The MDSFS-EMB feature selection algorithm screened features common to all datasets, which may result in the omission of features unique to some datasets, and reduce the personalized prediction ability of the model;
- The study only focused on BP values without incorporating demographic data (age, gender) and physiological state parameters (exercise/resting state), which may overlook covariates of BP fluctuations, and future multimodal feature fusion models need to be constructed to improve interpretability;
- The study did not explicitly investigate the interpretability of the model, especially its implications for real-world clinical applications, which represents a potential limitation for practical medical deployment.

## 5. Conclusions

In this study, a multi-source causal feature selection algorithm, the MDSFS-EMB algorithm, was proposed based on MB theory and the concept of causal invariance, which realized consistent causal feature screening across datasets. The algorithm collaborated with HITON-MB and PPFS to construct candidate MBs and defined the upper and lower bounds of causal feature search. Then, the features were ranked according to the average of mutual information. Finally, the causal invariant feature set across datasets was constructed by combining with GCMI, which demonstrated superior performance across three heterogeneous datasets compared to directly using the intersection or union of features from other methods. Moreover, it maintained robust predictive accuracy when applied to an independent validation dataset. This study explores the causal relationship between PPG-derived features and BP in a multi-source setting. By proposing a causal invariance-based multi-source feature selection framework, it provides a new solution for real-time BP monitoring in wearable medical devices.

## Figures and Tables

**Table 1:** Information about the datasets.

Dataset	Signal	Subject Number	Age Range	Subject’s Health Status
ABP_PPG	PPG	1195	57.1 ± 14.2	The subjects presented with a diverse array of medical conditions and were all intensive care unit patients.
	ABP			
BBD	PPG	40	34.2 ± 14.5	Four participants were diagnosed with cardiovascular-related diseases.
	ABP			
PPG-BP	PPG	219	56.9 ± 15.8	The subjects exhibited a diverse range of cardiovascular and related disorders.
	BP			
MS_PPG_BP	PPG	30	25 ± 1	Subjects were all healthy youths.
	BP			

**Table 2:** List of PPG features.

No.	Feature	Definition
1	Frequency Domain Features	FFT peak of the signal
2		Height of FFT peak
3		Mean value near the peak of the FFT
4–30	Time Domain Features	Point-of-interest and their features of PPG, VPG, APG, JPG, SPG
31–58		Time intervals
59–70		Areas under the PPG curve
71–91		Cyclicality
92–112		SW and DW at 25%, 50%, and 75% of the systolic peak amplitude 25%, 50%, and 75% of the systolic peak amplitude
113–114	Statistical Features	SQI: SQI-skew, SQI_kurtosis
115–118		Indices features
119–143		The extraction of Deviation Curve-based features
144–218		Histogram features of PPG, APG, VPG, JPG, SPG

**Table 3:** Comparison of model performance under different segmentation strategies.

Splitting Strategy	BP	MAD (mmHg)	STD (mmHg)	ME (mmHg)
sample-level splitting	DBP	2.91	4.16	−0.03
	SBP	4.70	6.67	−0.23
record-level splitting	DBP	5.42	6.11	−0.16
	SBP	8.34	10.56	−0.11

**Table 4:** Comparison of MAE and MASE under different feature selection methods.

Algorithm	BP	Number of Features	ABP_PPG		BBD		MS_PPG_BP		PPG-BP	
			MAD	MASE	MAD	MASE	MAD	MASE	MAD	MASE
∪PPFS	DBP	105	8.63	1.044	6.60	0.834	4.89	0.653	7.85	0.887
	SBP	103	12.08	0.686	9.68	0.787	5.73	0.450	11.96	0.730
∪HITON-MB	DBP	33	8.21	0.993	6.61	0.835	4.72	0.631	7.62	0.861
	SBP	35	11.33	0.643	9.82	0.798	6.36	0.499	9.57	0.584
∪*TREE*	DBP	120	8.67	1.048	6.60	0.834	4.88	0.652	7.03	0.794
	SBP	98	11.76	0.668	9.60	0.780	5.52	0.4336	10.58	0.650
∩PPFS	DBP	7	8.1	0.979	7.78	0.983	4.89	0.653	5.49	0.620
	SBP	2	11.66	0.6621	11.40	0.927	5.87	0.461	9.36	0.571
∩HITON-MB	DBP	1	7.74	0.936	5.99	0.757	4.41	0.589	7.30	0.825
	SBP	/	/	/	/	/	/	/	/	/
∩*TREE*	DBP	2	7.78	0.940	6.84	0.864	5.06	0.676	7.34	0.829
	SBP	1	11.88	0.675	10.79	0.877	6.60	0.476	9.33	0.600
MDSFS-EMB	DBP	21	6.32	0.764	5.69	0.719	3.71	0.495	5.58	0.630
	SBP	20	9.87	0.560	8.10	0.658	4.47	0.351	8.56	0.523

**Table 5:** Performance of machine learning estimation algorithms.

Datasets	Models		DBP			SBP	
		MAD	STD	ME	MAD	STD	ME
ABP_PPG	SVR	6.59	7.59	0.4	9.35	11.45	0.12
	RF	7.29	8.54	0.32	10.01	11.85	−1.51
	LightGBM	6.32	7.64	0.55	9.87	10.56	−1.05
	XGBoost	7.09	8.96	0.28	9.66	10.99	−0.74
BBD	SVR	5.57	6.88	−1.49	8.11	10.19	−0.1
	RF	5.75	6.60	−0.02	8.36	10.46	−0.06
	LightGBM	5.69	6.99	0.0	8.10	10.23	−0.07
	XGBoost	5.42	6.11	−0.16	8.34	10.56	−0.11
MS_PPG_BP	SVR	3.61	5.32	0.02	5.54	8.48	−0.86
	RF	3.76	5.50	0.09	4.76	7.43	−0.22
	LightGBM	3.71	5.48	0.06	4.47	7.24	−0.20
	XGBoost	3.19	4.43	0.07	4.75	7.05	−0.40
PPG	SVR	6.34	8.63	−0.32	8.98	11.49	−1.16
	RF	5.49	8.38	0.13	8.82	11.66	−0.23
	LightGBM	5.58	8.86	0.20	8.56	11.42	0.04
	XGBoost	5.58	8.89	0.06	8.04	11.98	−0.61

## Data Availability

The data presented in this article are available upon request from the corresponding author.

## Abbreviations

The following abbreviations are used in this manuscript:

AAMI	Association for the Advancement of Medical Instrumentation
ABP	Arterial blood pressure
AMI	Average Mutual Information
APG	Acceleration Photoplethysmography
BP	Blood Pressure
CVD	Cardiovascular diseases
DBP	Diastolic Blood Pressure
GCMI	Gaussian Copula Mutual Information
JPG	Jerk Photoplethysmography
MAD	Mean Absolute Difference
MASE	Mean Absolute Scaled Error
MB	Markov Blanket
ME	Mean Error
MDSFS-EMB	Multi-Dataset Stable Feature Selection via Ensemble MB
MS_PPG_BP	Multi-state PPG Blood Pressure Dataset
PPG	Photoplethysmography
RF	Random Forest
SBP	Systolic Blood Pressure
SDE	Standard Deviation of Error
SPG	Snap Photoplethysmography
SQI	Signal Quality Index
SVR	Support Vector Regression
SW and DW	Widths of the systolic and diastolic phases
VPG	Velocity photoplethysmography
